# Identifying Factors Associated with Risk Assessment Competencies of Public Health Emergency Responders

**DOI:** 10.3390/ijerph14060597

**Published:** 2017-06-04

**Authors:** Jiejing Hao, Jiaojiao Ren, Qunhong Wu, Yanhua Hao, Hong Sun, Ning Ning, Ding Ding

**Affiliations:** 1 Department of Social Medicine, School of Public Health, Harbin Medical University, Harbin 150000, China; haojiejing@126.com (J.H.); kelly_ren@163.com (J.R.); hyhyjw@126.com (Y.H.); sunhong_1965@aliyun.com (H.S.); dingdingmail0000@126.com (D.D.); 2 The Innovation Center for Social Risk Governance in Health, Shanghai 200032, China

**Keywords:** public health emergency, Center for Disease Control and Prevention (CDC), risk assessment

## Abstract

This study aimed to better understand the current situation of risk assessment and identify the factors associated with competence of emergency responders in public health risk assessment. The participants were selected by a multi-stage, stratified cluster sampling method in Heilongjiang Centers for Disease Control and Prevention (CDC). The questionnaires that measured their perceptions on risk assessment competences were administered through the face-to-face survey. A final sample of 1889 staff was obtained. Of this sample, 78.6% of respondents rated their own risk assessment competences as “relatively low”, contrasting with 21.4% rated as “relatively high”. Most of the respondents (62.7%) did not participate in any risk assessment work. Only 13.7% and 42.7% of respondents reported participating in risk assessment training and were familiar with risk assessment tools. There existed statistical significance between risk assessment-related characteristics of respondents and their self-rated competences scores. Financial support from the government and administrative attention were regarded as the important factors contributing to risk assessment competences of CDC responders. Higher attention should be given to risk assessment training and enhancing the availability of surveillance data. Continuous efforts should be made to remove the financial and technical obstacles to improve the competences of risk assessment for public health emergency responders.

## 1. Introduction

Following the landmark events of the Severe Acute Respiratory Syndromes (SARS) outbreak, the rapid expansion of H1N1 worldwide, as well as frequent emergencies occurring in last decade, the international community has been brought into a high-risk society threatened by public health emergencies. Risk assessment serves as an effective skill of health emergency response professionals, and has gained more and more attention globally [1]. The advantage of risk assessment skills of public health emergency responders lies in the ability to identify and describe future events that can be mitigated or prevented by long-term and strategic risk reduction measures [2]. In 2014, the epidemic of Ebola virus disease (EVD) in West Africa brought a great disaster to the world and caused irreparable loss, partly due to the lack of local risk management mechanisms and sufficient risk preparedness [3]. By contrast, a series of preventive measures such as assessing the potential risk of Ebola virus importation from West Africa into China and promptly making a risk preparedness plan and taking various preventive measures were implemented by the Chinese government, which, at the most, prevented EVD from being imported and causing an outbreak in China [4].

Public health emergency risk assessment is a systematic process of defining and describing hazards by characterizing their probability, frequency, and severity, as well as evaluating adverse consequences [5]. It evaluates the risk of public health emergencies through risk identification, risk analysis, and risk evaluation, and also makes risk management suggestions [6]. Many developed countries such as U.S., Germany, Australia, and other countries regard risk assessment as the key link to deal with public health emergencies and have formed a set of mature operation processes and risk management systems [7,8,9]. Since the SARS epidemic, China has established a direct reporting system for a nationwide information network of infectious diseases. Within this system, primary care institutions and hospitals are all required to report two types of Class A infectious diseases, 26 types of Class B and 11 types of class C infectious diseases to local Centers for disease control and prevention (CDCs); after verification, local CDCs are required to submit such instances to the national CDC. The Chinese government has become increasingly aware of the importance of risk management for public health emergencies [10]. Regulation of public health emergencies risk assessment, issued by Ministry of Health in 2012, provided mandatory requirements that Centers for disease control and prevention (CDCs) are responsible for the event of public health risk surveillance and assessment [6]. Additionally, the Chinese government also has shifted the focus from a passive public health emergency response to a more proactive, earlier stage risk management. Therefore, public health professionals working in CDCs have become the forefront implementers of risk assessment, and their competencies in risk assessment has important bearing on the prevention and control of public health emergencies [11]. To better understand the current situation of public health risk assessment in China, this study, from the perspective of self-rated competences by professional staff of CDCs, aimed to find out the needs of the public health emergency responders and to explore factors associated with risk assessment competences, which can provide evidence on how China may better improve its public health emergency management system.

## 2. Methods

### 2.1. Study Population and Data Collection

A face-to-face survey was carried out by researchers from the department of Social Medicine of Harbin Medical University in Heilongjiang province. Heilongjiang is a province located in northeast China with 13 jurisdictional regions and 174 CDC agencies with a total of 5450 professionals, according to the data from the health statistics yearbook of Heilongjiang province in 2011 [12]. Between 2004 and 2012, Heilongjiang reported 12,587 epidemic cases and 55 related deaths. These public health emergencies put a huge burden on the development of the local regions [13]. This made the local government fully realized that the most cost-effective intervention of public emergencies would be to prioritize risk management, which also answered the urgent call from the central government to strengthen public health risk assessment.

A pilot study was conducted with 20 public health emergency responders prior to the formal survey, in order to test the research design. Then, two focus groups were organized with 10 participants for each group from four local CDCs. First, they were invited to fill out the questionnaires by themselves, and then their comments and opinions were solicited relating to the appropriateness of the questionnaire design, as well as any improper questions and answers. Afterwards, some necessary adjustments were made in light of the responses.

The full-scale research survey was then conducted. A stratified cluster sampling method was adopted. The study included all 13 municipalities in Heilongjiang and two to four counties of each municipality at random, in consideration of the geographical and jurisdictional diversity. Meanwhile, we asked for CDC administrators’ willingness to take part in this survey for further cooperation and support. Finally, 40 CDCs were sampled. All employees of the 40 CDCs were invited to participate in the survey, except for the non-professional staff, and the structured questionnaires were administered through face-to-face surveys. Quality control was implemented by supervisors throughout the study. The study protocol was reviewed and approved by the Research Ethics Committee of Harbin Medical University. All participants gave written, informed consent. In total, 1889 individuals completed the questionnaire.

### 2.2. Variables

The survey questionnaire involved basic demographic information, the participants’ perceived competences of risk assessment, the experience of participation in risk assessment in public health emergencies, the degree of familiarity with risk assessment tools and methods, and the factors associated with risk assessment competence of public health emergency responders. The question ‘How would you rate your risk assessment competence?’ was identified as the dependent variable to measure self-rated risk assessment competence from public health emergency responders. Responses were rated on an ordinal scale (1 = ‘very poor’, 2 = ‘poor’, 3 = ‘neither poor nor good’, 4 = ‘good’, 5 = ‘very good’). Because the distribution of the dependent variable showed a slightly negative skew with a mean of 2.51 and a median of 3, the responses were grouped into two categories with a midpoint of 3: ‘low competence’ and ‘high competence’ (the first three items were grouped as ‘low competence’ and the latter two were grouped as ‘high competence’).

Independent variables included demographic information such as gender, age, education background, professional title, and work experience. Participants were asked to rate their own mastery of key techniques required throughout the whole process of risk evaluation from the following questions: ‘How proficient are you in making a risk assessment plan? How proficient are you in local public health risk identification? How proficient are you in risk analysis? How proficient are you in risk evaluation? How proficient are you in risk treating? How proficient are you in risk assessment report writing?’ Responses were rated on an ordinal scale from 1 to 5 (1 = ‘not at all proficient’, 2 = ‘slightly proficient’, 3 = ‘moderately proficient’, 4 = ‘very proficient’, 5 = ‘extremely proficient’). All the respondents were asked what (if any) risk assessment work they had participated in. Meanwhile, the respondents were asked how familiar they were with the following risk assessment tools and methods: likelihood analysis, consequence analysis, expert judgment, risk matrix, vulnerability analysis, and risk assessment software, which were recommended by the World Health Organization (WHO) in regular risk assessment. As for the factors associated with risk assessment competence, the respondents scored the importance from 1 to 5, including financial support, risk assessment tools, senior leaders’ attention, authority requirements, severity of historical emergency events, risk assessment staffing, risk assessment techniques, frequency of occurred emergencies, risk assessment training and drill, and availability of surveillance data.

### 2.3. Statistical Analysis

Descriptive statistics were calculated to describe the demographic characteristics. The variables that demonstrated statistical significance (*p* < 0.05) by the bivariate analysis were analyzed by the multivariate logistic regression to predict the outcome of the self-rated risk assessment competence of public health emergency responders. Odds Ratio (OR) and their 95% Confidence Interval (CI) were estimated to assess the relationship between the predictors and the outcome variable. All statistical analyses were performed using IBM SPSS Statistics for Windows, Version 22.0. (IBM Corp, Armonk, NY, USA).

## 3. Results

### 3.1. The Perceived Competencies of Risk Assessment with Characteristics of Study Participants

Of the 1889 CDC respondents (Table 1), 41.1% were male and 58.9% were female. About half of the respondents (51.5%) were more than 40 years of age, and the majority (54.3%) had worked less than 15 years. Further, 46.0% of respondents reported having college or graduate qualifications. Only 20% of respondents had senior professional titles.

Of the study sample, 78.6% rated their own risk assessment competence as “relatively low”, contrasting with 21.4% rated as “relatively high”. Except for education background, there existed significant differences in all sociodemographic characteristics on the self-rated general competence. Those who were senior employees, with a higher professional title, with public health risk assessment experience, trained in risk assessment and familiar with risk assessment tools, tended to have a relatively higher self-assessment. Multivariate analysis results showed that there were statistically significant correlations between risk assessment-related characteristics and perceived competencies (Table 2).

### 3.2. The Perceived Competience in Key Processes of Risk Assessment

Associations calculated indicated that there existed great differences between CDC respondents with risk assessment–related characteristics and the self-rated scores of key processes of risk assessment (Table 3). Respondents with training experience in risk assessment had the highest score in all of the six risk assessment processes, and those who were unfamiliar with risk assessment tools had the lowest score. Among those with increased age, length of employment, and promotion in title, the self-rated competence scores of respondents in risk assessment tended to be relatively high. In comparison, respondents had higher scores in risk identification and lower scores in making risk assessment plans. Although there was no statistically significant correlation between individual education background and the competence scores in the other five risk assessment processes, better educated respondents showed relatively high competence in risk assessment report writing.

### 3.3. Assessment on Different Public Health Risks

**Figure 1:**
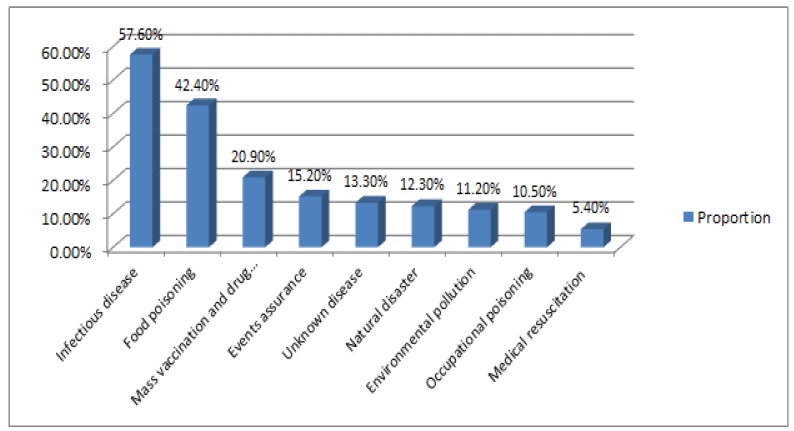
The proportion of different public health risks assessed by respondents (%).

Overall, the risk assessment work in public health emergencies in CDCs was not carried out well. Most of the respondents (62.7%) did not participate in any risk assessment work. As shown in Figure 1, of 705 respondents who had ever undertaken risk assessment, 57.6% was related to infectious disease risks and 42.4% was related to food poisoning risks. Mass vaccination and drug response-related risk assessment accounted for 20.9%. The remaining proportions of assessment covered public health risks arising from events assurance, unknown disease, natural disaster, environmental pollution, and occupational poisoning were less than 20%. Medical resuscitation-related risk assessment was at the lowest proportion (5.4%).

### 3.4. Respondents’ Familiarity with Risk Assessment Tools and Methods

**Figure 2:**
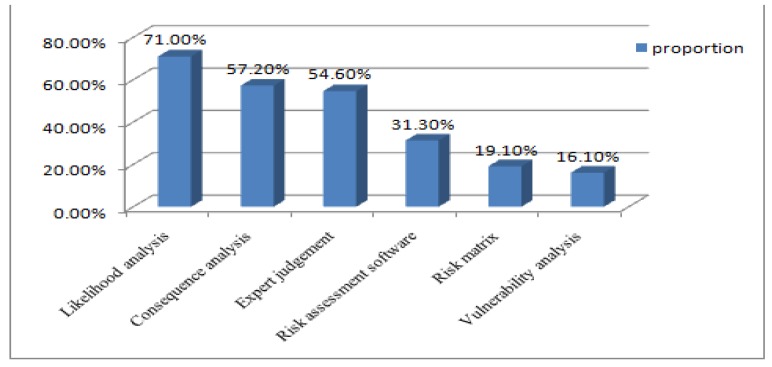
The proportion of respondents’ familiarity with risk assessment tools (%).

42.7% of CDC respondents reported that they were familiar with risk assessment tools which were recommended by the WHO in regular risk assessment. Among these tools and risk assessment methods, likelihood analysis was well known to 71% of respondents, followed by consequence analysis with 57.2% of respondents. 54.6% were familiarized with expert judgment in risk analysis. Only 19.1% and 16.1% of respondents were familiar with risk matrix and vulnerability analysis (See Figure 2). In addition, 31.3% of respondents indicated that they were proficient in the use of risk assessment software.

### 3.5. Perceived Factors Associated with Risk Assessment Competences by Respondents

**Figure 3:**
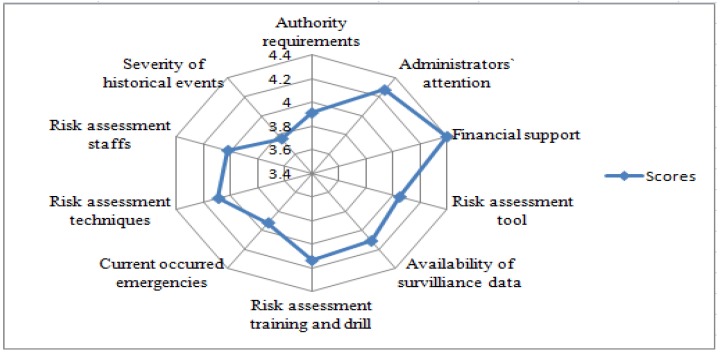
Importance scores of perceived factors associated with risk assessment competencies.

The respondents considered that there existed several factors associated with their risk assessment competencies (Figure 3). Financial support from the government was regarded as the most important factor, with the score of 4.40 ± 0.96, followed by the factor of administrators’ attention (4.27 ± 1.00). The scores of specialized training and risk assessment drills and availability of surveillance data were also more than 4.1. The other three factors including risk assessment technique, tools and methods, and workers, had the scores of 4.0. The severity of historical emergency events was deemed as a limited impact on risk assessment with the score of 3.76 ± 1.18.

## 4. Discussion

A major goal of the present study was to understand the competence of CDC respondents to carry out risk assessment and examine the associations between self-rated competence scores and characteristics of study participants including demographics and risk assessment-related issues, in order to further explore perceived factors that impact the risk assessment competencies of respondents, which may be instructive for the promotion of local public health emergencies management. These results are discussed along, with the limitations of the study.

### 4.1. The Weak Competencies of Risk Assessment Perceived by Participants in Public Health Emergencies

This study found that 78.6% of CDC respondents rated their own risk assessment competence as “relatively low”, contrasting with 21.4% rated as “relatively high”, with the average score of 2.51 out of a maximum of 5. Instead of the overestimation of self-assessment [14], the weak competence exhibited by the sampled province tended to be much more believable, which also consistent with the results of previous studies from other regions in China [15,16,17]. Ever since Regulation of public health emergencies risk assessment was issued by the Ministry of Health, public health risks assessment was required to be undertaken periodically. CDC respondents still lack adequate awareness of their own responsibility related to risk assessment. Further competency analysis of the six key processes of risk assessment showed that making risk assessment plans and writing reports had the lowest scores. The majority of CDC respondents held the idea that assessment plans should be made by the most responsible person; risk assessment reports should be written by a specially-assigned person as well. These tasks were considered outside of their responsibilities, and therefore the corresponding competencies were unnecessary. By contrast, the respondents’ competence of risk identification had the highest score. Risk identification is the critical first step of the risk management process, and respondents with risk assessment-related characteristics presented better abilities. In addition, those who were familiar with assessment tools could undertake risk analysis rather well. Moreover, with experience in risk assessment, more and more CDC respondents could carry out risk evaluation and risk treatment.

### 4.2. Significant Associations Between Risk Assessment-Related Characteristics of Respondents and Their Competence Score

Whether the respondents carried out risk assessment or not was a significant variable to their perceived competence. The results found that many respondents were not involved in risk assessment work. After the outbreak of SARS, China had set up independent health emergency departments and initiated a series of programs to strengthen the emergency system’s resilience to public health emergencies [18]. However, risk assessment work was still in its infancy. This showed that CDC respondents were to a large extent unaware of relevant theory and practice [19]. Nevertheless, the already launched risk assessment in Heilongjiang CDC covered various kinds of risks, from infectious disease risk at most to medical resuscitation risk at least, which was in accordance with the regular work in CDCs [20].

In addition, there existed a statistically significant correlation between the respondents’ familiarity with risk assessment tools and methods and their competence scores. Risk assessment tools helped CDC respondents assess and prioritize risk interventions based on the impact and the likelihood of the risk occurring [2]. With lower familiarity rate of 42.7%, CDC respondents need to strengthen the use and study of risk assessment tools according to national CDC technical guidance. Although they showed relatively high familiarity with analysis tools on likelihood, consequence of risk, and expert judgment, the most important and valuable tools of risk matrix and vulnerability analysis were less understood by CDC respondents in risk assessment. To address this issue, providing risk assessment training is an effective approach [21].

The results found that training was a key element for risk assessment competence in public health emergency responders. Through training, CDC respondents can understand key practical risk assessment techniques to identify hazards and evaluate risk, to know how to implement monitoring, and to know when and how to perform the review process [22]. However, only 259 out of 1889 respondents had participated in risk assessment training. Therefore, there is an urgent need to strengthen risk assessment training among CDC respondents, especially for those with junior positions, lower professional titles, and lack of public health risk assessment experience. It is necessary to establish a long-term mechanism for risk assessment training and to push forward training systematically [23].

### 4.3. The Contributory Factors to Risk Assessment Competencies of Public Health Emergency Responders

Financial support from the government was regarded as the most important factor that influenced the risk assessment competence of public health emergency responders. In China, CDCs usually receive a fixed budget from the local government. New task requirements from the central government might not necessarily be transferred into increased local government funding to CDCs, especially in provinces that face economic challenges. Sound risk assessment requires a great effort in risk-related information collection, surveying, and risk identification, as well as analysis on vulnerability at individual, organizational and systematic levels. Moreover, conducting risk evaluation and control also requires additional government input. Without sufficient government investment, risk assessment will only end with useless paperwork, which in turn will also demotivate the enthusiasm of CDC staff for improving their risk assessment skills [24].

Administrators’ attention was deemed as another important factor for the respondents’ competence to undertake risk assessment. Encouragement and attention from chief executives of health administrative departments or CDCs can effectively improve the performance of employees [25], which is different from work requirements and risk assessment techniques, especially in the current context of the administrative management system in China.

In addition, the availability of risk surveillance data was perceived as the other factor impacting the risk assessment competence of public health emergency responders. Data monitored and collected timely by the respondents was premier to risk assessment [26]. According to abnormal changes in risk data under surveillance, CDC respondents can effectively detect early signs of public health risks as soon as possible and put forward relevant suggestions [27].

This study has several limitations. The terminology and some questions associated with risk assessment may have caused unclear and vague responses from some participants, especially among those who lacked experience in risk assessment. However, the face-to-face survey made by trained surveyors minimized this potential bias. Due to limited resources, the survey was conducted only in Heilongjiang province; therefore, these findings cannot be generalized to other regions in China. Further studies are needed to develop a more comprehensive evaluation tool and enhance the risk assessment competence of public health emergency responders.

## 5. Conclusions

CDC respondents in Heilongjiang province revealed their own incompetence in risk assessment. Lack of experience in risk assessment, inadequate training, and little familiarity with risk assessment tools all had a significant impact on the weak competencies of CDC employees. Government financial support and administrative attention was regarded as the important factors contributing to the risk assessment competences of CDC responders. Higher attention should also be given to the availability of surveillance data. Strengthening risk assessment training, especially in the use of risk assessment tools and detailed operation of six key risk assessment processes, could be an effective approach to address these technical issues. Continuous efforts should be made to remove the financial and technical obstacles in order to improve the risk assessment competence of public health emergency responders.

## Figures and Tables

**Table 1:** Bivariate analysis between perceived competencies of risk assessment and characteristics of study participants.

Variable	n (% of 1889)	Low Competence n (% of 1485)	High Competence n (% of 404)	*p*-Value
Gender				
Male	776 (41.1)	588 (39.6)	188 (46.5)	0.014
Female	1113 (58.9)	897 (60.4)	216 (53.5)	
Age				
Less than or equal to 40 years old	917 (48.5)	742 (50.0)	175 (43.3)	0.010
More than 40 years old	972 (51.5)	743 (50.0)	229 (56.7)	
Education				
Junior college or below	1021 (54.0)	806 (54.3)	215 (53.2)	0.736
University or above	868 (46.0)	679 (45.7)	189 (46.8)	
Professional title				
Junior title or below	1511 (80.0)	1206 (81.2)	305 (75.5)	0.014
Senior title	378 (20.0)	279 (18.8)	99 (24.5)	
Working experience				
Less than or equal to 15 years	1026 (54.3)	821 (55.3)	205 (50.7)	0.058
More than 15 years	863 (45.7)	664 (44.7)	199 (49.3)	
Has carried out public health risk assessment				
No	1184 (62.7)	1041 (70.1)	143 (35.4)	0.000
Yes	705 (37.3)	444 (29.9)	261 (64.6)	
Has participated in risk assessment training				
No	1630 (86.3)	1342 (90.4)	288 (71.3)	0.000
Yes	259 (13.7)	143 (9.6)	116 (28.7)	
Familiar with risk assessment tools				
No	1083 (57.3)	952 (64.1)	131 (32.4)	0.000
Yes	806 (42.7)	533 (35.9)	273 (67.6)	

**Table 2:** Multivariate analysis between perceived competencies of risk assessment and characteristics of study participants.

Variables	Odds Ratio (95% CI)	*p*-Value
Gender (Male vs. Female)	0.856 (0.671 to 1.091)	0.210
Age (≤40 vs. >40)	1.249 (0.938 to 1.664)	0.128
Professional title (Junior title vs. Senior title )	1.265 (0.931 to 1.718)	0.133
Working experience (≤15 years vs. >15 years )	0.876 (0.652 to 1.175)	0.379
Carried out public health risk assessment (No vs. Yes)	2.966 (2.207 to 3.985)	0.000
Participated in risk assessment training (No vs. Yes)	1.781 (1.309 to 2.423)	0.000
Familiar with risk assessment tools (No vs. Yes)	1.691 (1.256 to 2.275)	0.001

**Table 3:** Bivariate analysis between self-rated scores on key processes of risk assessment and characteristics of study participants.

Characteristics of Study Participants	Plan Scores	Identification Scores	Analysis Scores	Evaluation Scores	Treatment Scores	Report Scores
Gender (Male vs. Female)	2.43 (1.29) vs. 2.24 (1.22) *	2.60 (1.28) vs. 2.44 (1.20) *	2.58 (1.29) vs. 2.37 (1.20) *	2.56 (1.30) vs. 2.36 (1.21) *	2.55 (1.30) vs. 2.35 (1.24) *	2.43 (1.29) vs. 2.26 (1.22) *
Age (≤40 vs. >40)	2.25(1.23) vs. 2.38 (1.27) *	2.43 (1.21) vs. 2.58 (1.25) *	2.39 (1.24) vs. 2.52 (1.24) *	2.38 (1.24) vs. 2.50 (1.26) *	2.38 (1.24) vs. 2.49 (1.29)	2.28 (1.23) vs. 2.37 (1.28)
Education (Junior college vs. University)	2.29 (1.26) vs. 2.35 (1.25)	2.50 (1.24) vs. 2.51 (1.23)	2.44 (1.25) vs. 2.49 (1.22)	2.42 (1.25) vs. 2.47 (1.25)	2.41 (1.27) vs. 2.46 (1.25)	2.24 (1.24) vs. 2.43 (1.27) *
Professional title (Junior title vs. Senior title)	2.28 (1.23) vs. 2.48 (1.32) *	2.47 (1.23) vs. 2.66 (1.25) *	2.42 (1.25) vs. 2.60 (1.21) *	2.41 (1.25) vs. 2.58 (1.26) *	2.40 (1.26) vs. 2.58 (1.27) *	2.28 (1.24) vs. 2.53 (1.29) *
Working experience (≤15years vs. >15 years)	2.21 (1.22) vs. 2.44 (1.28) *	2.43 (1.24) vs. 2.59 (1.23) *	2.38 (1.24) vs. 2.55 (1.23) *	2.36 (1.24) vs. 2.54 (1.25) *	2.36 (1.25) vs. 2.52 (1.28) *	2.26 (1.24) vs. 2.41 (1.27) *
Has carried out public health risk assessment (Yes vs. No)	3.05 (1.12) vs. 1.88 (1.11) *	3.21 (1.03) vs. 2.09 (1.16) *	3.16 (1.06) vs. 2.04 (1.15) *	3.16 (1.08) vs. 2.02 (1.15) *	3.15 (1.09) vs. 2.01 (1.17) *	3.07 (1.12) vs. 1.89 (1.12) *
Has participated in risk assessment training (Yes vs. No)	3.25 (1.04) vs. 2.17 (1.22) *	3.32 (1.02) vs. 2.38 (1.22) *	3.34 (0.99) vs. 2.32 (1.22) *	3.33 (0.98) vs. 2.30 (1.23) *	3.32 (1.03) vs. 2.29 (1.24) *	3.24 (1.10) vs. 2.18 (1.22) *
Familiar with risk assessment tools (Yes vs. No)	2.97 (1.11) vs. 1.83 (1.13) *	3.15 (0.99) vs. 2.03 (1.18) *	3.15 (1.03) vs. 1.94 (1.13) *	3.14 (1.05) vs. 1.92 (1.13) *	3.11 (1.05) vs. 1.93 (1.17) *	2.99 (1.10) vs. 1.84 (1.13) *
All participants	2.32 (1.25)	2.51 (1.24)	2.46 (1.24)	2.44 (1.25)	2.44 (1.27)	2.33 (1.25)

* Statistically significant at 0.05.

## References

[1] Liu J. (2008). The Introduction to Risk Management.

[2] Jones T. (2008). Advances in risk assessment for Australian emergency management. Aust. J. Emerg. Manag..

[3] Xu T. (2015). The War on Ebola: Crises, challenges, and implications. J. Int. Stud..

[4] Huang Y. (2017). China’s response to the 2014 Ebola outbreak in West Africa. Glob. Chall..

[5] World Health Organization Rapid Risk Assessment of Acute Public Health Events. http://www.who.int/csr/resources/publications/HSE_GAR_ARO_2012_1/en/.

[6] Ministry of Health (2012). Regulation of Public Health Risk Assessment in Emergencies.

[7] U.S. Department of Homeland Security (2007). Target Capabilities List: A Companion to the National Preparedness Guidelines.

[8] Federal Office of Civil Protection and Disaster Assistance Method of Risk Analysis for Civil Protection. https://www.zki.dlr.de/research.

[9] Emergency Management Australia (2004). Emergency Risk Management Applications Guide Manual 5.

[10] Wang L. (2008). The Emergency Management of Public Health Emergencies: Theory and Practice.

[11] Hu G., Rao K., Sun Z. (2007). Investigation into the capacity for risk identification, assessment, and mitigation in managing public health emergencies in China. Acta Acad. Med. Sin..

[12] Bureau of Health in Heilongjiang (2011). Health Statistics Yearbook in Heilongjiang Province.

[13] Yan S., Wang X., An Y., Gao Y. (2013). Epidemiology analysis on the public health emergencies from 2004 to 2012 in Heilongjiang Province. Chin. J. Public Health Manag..

[14] Kerby D., Brand M., Johnson D. (2005). Self-assessment in the measurement of public health workforce preparedness for bioterrorism or other public health disasters. Public Health Rep..

[15] Bu L., Hao X., Xu M., Wu J., Liu J., Xu L., Ta N., Huang L. (2014). Comparative cognitive study of public health emergency risk assessment among emergency professionals. Chin. Health Resour..

[16] Liu Z., Hao X., Zhang Z., Wu J., Xu M., Liu J., Gao Y., Xu L., Ta N. (2013). Cognitive appraisal of public health emergency risk assessment among China’s provincial emergency personnel. Chin. Hosp. Manag..

[17] Sun Y., Hao Y., Wu Q., Jiao M., Kang Z., Huang K., Ma J., Peng L. (2013). Study on risk assessment capacity and the influencing factors of health emergency personnels in Heilongjiang province. Chin. J. PHM.

[18] Ministry of Health (2006). Construction Planning of the National Public Health Emergency Response System During “11th Five-Year”.

[19] Deng X., Chen G., Huang Y., Chu D., Gao Y. (2014). Risk assessment method for public health emergencies. Chin. Prev. Med..

[20] Ministry of Health (2007). Regulation on Responsibility of Public Health Emergency in Chinese Centers for Disease Control and Prevention.

[21] World Health Organization Public health Risk Assessment and Interventions: The Libyan Arab Jamahiriya. http://www.who.int/diseasecontrol_emergencies/publications/who_hse_gar_dce_2011_1/en/.

[22] Federal Emergency Management Agency Emergency Management Institute. Unit 11: Course Training. http://www.training.fema.gov/EMIWEB.

[23] Xu Y., Wu Q., Lin T., Shang J. (2009). The strategy choosing of public health emergencies training overseas and the guiding significance to China. Chin. Health Econ. J..

[24] Luo Y., Zhang L. (2013). Current situation and problems of emergency management funds in China. Dig. Manag. Sci..

[25] Yanrui L., Yanhua H., Qunhong W. (2010). Study on the current situation of performance appraisal and analysis of existing problems in Center for Disease Control and Prevention. Chin. Health Econ. J..

[26] Zhang Y., Chen Q., Chen S., Yang Y., Zou Z., Li L., Xu Y. (2007). A primary evaluation of public health emergency surveillance and early-warning in medical institutions in Guangdong. J. Guangdong Coll. Pharm..

[27] Qunhong W., Zheng K., Mingli J. (2014). Theory and Technical Guidance for Risk Assessment of Public Health Emergencies.

